# Dense module searching for gene networks associated with multiple sclerosis

**DOI:** 10.1186/s12920-020-0674-5

**Published:** 2020-04-03

**Authors:** Astrid M. Manuel, Yulin Dai, Leorah A. Freeman, Peilin Jia, Zhongming Zhao

**Affiliations:** 1 Center for Precision Health, School of Biomedical Informatics, The University of Texas Health Science Center at Houston, 7000 Fannin St. Suite 600, Houston, TX 77030 USA; 2 Department of Neurology, McGovern Medical School, The University of Texas Health Science Center at Houston, Houston, TX 77030 USA; 3 Human Genetics Center, School of Public Health, The University of Texas Health Science Center at Houston, Houston, TX 77030 USA; 4 Department of Biomedical Informatics, Vanderbilt University Medical Center, Nashville, TN 37203 USA

**Keywords:** GWAS, Multiple sclerosis, dmGWAS, Network module, Gene set enrichment analysis, Drug target

## Abstract

**Background:**

Multiple sclerosis (MS) is a complex disease in which the immune system attacks the central nervous system. The molecular mechanisms contributing to the etiology of MS remain poorly understood. Genome-wide association studies (GWAS) of MS have identified a small number of genetic loci significant at the genome level, but they are mainly non-coding variants. Network-assisted analysis may help better interpret the functional roles of the variants with association signals and potential translational medicine application. The Dense Module Searching of GWAS tool (dmGWAS version 2.4) developed in our team is applied to 2 MS GWAS datasets (GeneMSA and IMSGC GWAS) using the human protein interactome as the reference network. A dual evaluation strategy is used to generate results with reproducibility.

**Results:**

Approximately 7500 significant network modules were identified for each independent GWAS dataset, and 20 significant modules were identified from the dual evaluation. The top modules included *GRB2, HDAC1*, *JAK2*, *MAPK1,* and *STAT3* as central genes. Top module genes were enriched with functional terms such as “regulation of glial cell differentiation” (adjusted *p*-value = 2.58 × 10^− 3^), “T-cell costimulation” (adjusted *p*-value = 2.11 × 10^− 6^) and “virus receptor activity” (adjusted *p*-value = 1.67 × 10^− 3^). Interestingly, top gene networks included several MS FDA approved drug target genes *HDAC1*, *IL2RA*, *KEAP1*, and *RELA,*

**Conclusions:**

Our dmGWAS network analyses highlighted several genes (*GRB2, HDAC1, IL2RA, JAK2, KEAP1, MAPK1, RELA* and *STAT3*) in top modules that are promising to interpret GWAS signals and link to MS drug targets. The genes enriched with glial cell differentiation are important for understanding neurodegenerative processes in MS and for remyelination therapy investigation. Importantly, our identified genetic signals enriched in T cell costimulation and viral receptor activity supported the viral infection onset hypothesis for MS.

## Background

Multiple sclerosis (MS), a chronic and disabling disorder of the central nervous system (CNS), affects nearly 1 million individuals in the United States [1]. MS is characterized by inflammation, demyelination, defective remyelination and neuronal injury that may be extensive, leading to permanent and progressive disability. MS is typically diagnosed between 20 and 40 years of age, and is nearly three times more frequent in women than in men [1, 2]. The cause of MS remains unknown, and it is unclear if its etiology may even vary from patient to patient. MS is thought to occur in genetically predisposed individuals with potential trigger, or at least contribution of environmental factors [3]. One hypothesis involves a yet unknown CNS viral infection triggering T-cell auto-reactivity through a mechanism of molecular mimicry [4]. In light of the high prevalence of the disease and its poorly elucidated mechanism, it is of importance to further study MS pathogenesis.

Studying genetic factors associated with MS may aid in understanding its pathophysiology. The realization that MS risk is significantly higher for family members of affected individuals has prompted a search for genetic predisposing factors [5]. Early genetic studies of MS developed genetic mapping for families affected with MS, which identified human leukocyte antigen (HLA) regions to be associated with MS [5, 6]. Due to limitations of sample size, these early studies were ineffective in validating other genetic risk factors of MS [5]. Pivotally, genome-wide association studies (GWAS), which during recent years have aided in acquiring vast repositories of genetic variations associated with specific phenotypes, were performed in large MS populations [7]. As GWAS were performed for larger MS sample sizes than original genetic mapping studies, several genetic variations, or single nucleotide polymorphisms (SNPs), outside of the HLA region became attributed to MS [8, 9]. Some of these MS GWAS studies are publicly available in the form of GWAS summary statistics datasets, which summarize SNP level association *p* values in the specific population or subpopulation (i.e., without individual level genotype information to the public) [7]. In this work, two publicly available MS GWAS summary statistics datasets are independently and conjunctively further studied: a MS GWA study performed by the Genetic Multiple Sclerosis Association (GeneMSA) consortium in 2009 and a MS GWA study performed by the International Multiple Sclerosis Genetics Consortium (IMSGC) in 2011 [8, 9]. Both of these GWA studies reported some SNPs with genome-wide significance levels, but these variants are largely in the non-coding regions with unknown function, making biological interpretation difficult.

Although GWAS have come a long way in elucidating genetic variations associated with specific traits, integrating GWAS data with other kinds of datasets can aid in understanding the molecular underpinnings behind these associations [10, 11]. Additionally, differing genotyping platforms used by GWAS and other factors (ethnic background, sample size, statistical tests) can make it difficult to perform meta-analytical studies, but network-based analysis is a promising approach to detect combinatory association signals in network modules. This study aims to apply network approach to find joint association signals at the network modules, and thus, leading to biologically interpretable results [12, 13]. A new version of the dense module searching algorithm (dmGWAS) is used to integrate GWAS signals with a comprehensive human protein-protein interaction (PPI) network so that MS candidate subnetworks can be identified [11]. Uniquely, dense module searching was performed independently on the 2 MS GWAS studies mentioned above. Lastly, this study also aims to jointly examine independent MS GWAS modules by using the dual evaluation function of the dmGWAS package. This evaluation strategy would enhance the results in a reproducible fashion, since the replication rate is often low in GWA studies of complex disease.

## Materials and methods

### Obtaining gene-level *p*-values by Pascal

The GeneMSA GWAS and IMSGC GWAS summary statistics data were respectively accessed through the public databases dbGaP and GWAS Catalogue [14, 15]. The human PPI network was downloaded from the public Human Protein Reference Database (HPRD) [16], as demonstrated in [17, 18]. The GeneMSA GWAS data was collected by the Sentrix HumanHap 550-BeadChip genotyping platform, and the IMSGC GWAS data was collected by the Human660-Quad chip genotyping platform [8, 9] (Additional file 1: Table S1).

**Fig. 1:**
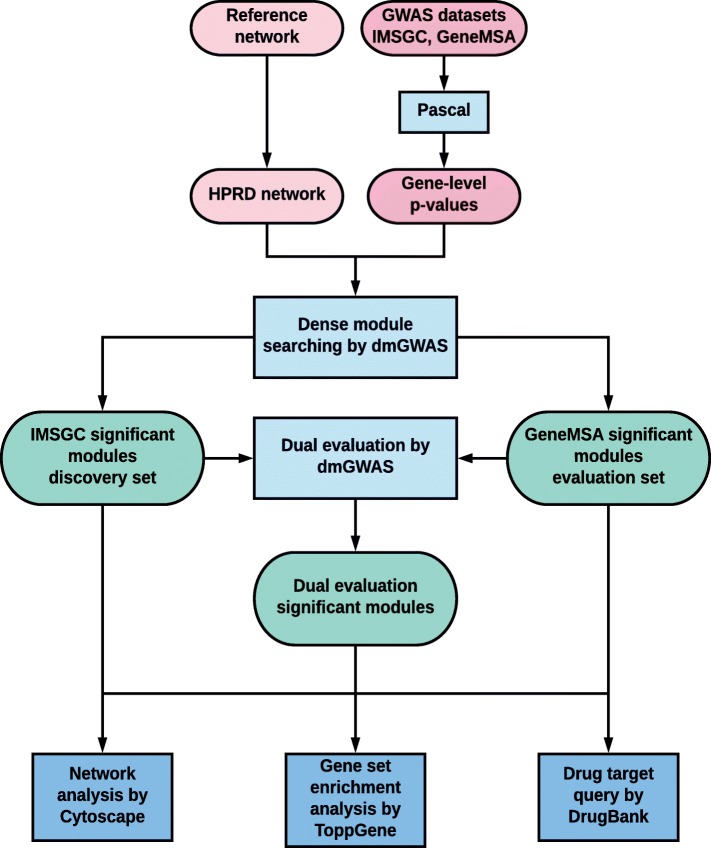
Pipeline for dmGWAS implementation and gene network assessment. Details are provided in the Materials and methods section

Summary statistics data is presented in the form of SNP-level association *p*-values. In this study, gene-level *p*-values (p_g_) are needed as input for dense module searching by dmGWAS. To obtain p_g_ measures, a pathway scoring algorithm (Pascal) was used, which considers combined SNP-level effects (Fig. 1) [19]. The p_g_ values were obtained with default Pascal settings, which uses the sum-of-chi-squares (SOCS) test and considers the gene region along with 50 kilobase pairs upstream/downstream of the gene region [19]. Pascal p_g_ measurements were obtained independently for each MS GWAS dataset examined. We excluded those genes whose p_g_ was smaller than 10^− 12^ because they would likely be dominant in the top modules and disrupt our dense module searching process.

### Dense module searching (DMS) and dual evaluation by dmGWAS

Dense module searching was performed with the DMS function of dmGWAS (version 2.4) Linux binary R package that is available at [20]. This function calculates each p_g_ value as a node weight and scores each subgraph based on a quantitative summary of all the weights in each module [11]. This function was executed for each MS GWAS dataset independently. The outcome of DMS is in the form of an RData results file which contains modules with significant z-scores (Z_m_), where each module is named by its seed gene. Z_m_ measurements by dmGWAS are acquired by

$$ {Z}_m=\frac{\sum {Z}_i}{\sqrt{k}}, $$

where *z*_*i*_ was transferred from p_g_ following the normal distribution function, and k represents the number of genes present within each module [11]. The top modules with the highest Z_m_ values were considered for our independent network analysis and validation.

The dual evaluation function of dmGWAS (*dualEval*) takes into consideration two module output lists (each from a GWAS dataset in this analysis) from DMS function and performs dense module searching based on the two module lists (Fig. 1) [11]. This was used to assess the overall results of dense module searching from both GeneMSA GWAS and the IMSGC GWAS datasets. Specifically, dual evaluation was performed by using one GWAS dataset (e.g., IMSGC) as the discovery set and the other (e.g., GeneMSA) as the evaluation set, and the consistent results will be reported. Because the IMSGC GWAS had a larger sample size and stronger association signals, we used its DMS modules for the discovery (Additional file 2: Figure S1) and used the DMS modules from GeneMSA GWAS dataset to evaluate IMSGC modules. After implementation of the dualEval function by dmGWAS, significant modules from discovery set were merged.

### Network visualization and validation

The software Cytoscape was used for network visualization. Cytoscape is a popular open source tool commonly used for analysis of biomedical networks [21]. Nodes and edges derived from the module results by running dmGWAS version 2.4 were imported into Cytoscape. The NetworkAnalyzer tool, a built-in Cytoscape tool that measures a variety of network parameters, was used to assess the resulting networks [21]. The measure of betweenness and centrality (Betweenness) was used to measure the functional importance of the nodes in top modules. The betweenness value, computed by the Brandes fast algorithm, highlights nodes that join communities of dense subnetworks together [22–24]. Brandes fast algorithm computes betweenness by Freeman’s general formula:

$$ Betweenness=\sum \limits_{s\ne v\ne t\in V}\frac{\sigma_{st}(v)}{\sigma_{st}}, $$

where *V* is the set of vertices, *s* and *t* represent vertices such that *σ*_*st*_ can denote a path from *s* to *t*, and *σ*_*st*_(*v*) can denote the number of shortest paths from *s* to *t* that vertex *v* lies on [23, 25].

Functional enrichment analysis by ToppGene Suite [26] was performed for validation of our resulting MS gene networks. ToppGene (also known as ToppCluster) is a web-based tool that performs enrichment analysis based on annotations of experimentally validated categorical data about human genes [26]. Annotations from the Gene Ontology (GO) database were reported from our analysis [27]. Genes within top 10 modules were considered as the input for each functional enrichment analysis performed on individual GWAS data sets. The complete discovery set of genes from significant dual evaluation modules were considered for the functional enrichment analysis of the dual evaluation results. Calculations of significance level by ToppGene Suite was done by a probability density function [26]. Multiple testing correction was conducted using the Benjamini-Hochberg method for all annotation features, which provided adjusted *p*-values for enrichment results [28]. A functional enrichment annotation limit of 100 genes was chosen per annotation report, as all gene lists ranged from 26 to 56 genes.

A drug target search within the DrugBank database was performed for further evaluation of the translational potential of the resulting gene networks. DrugBank is a public online database, which contains rich biochemical information about drugs and their gene targets [29]. The DrugBank database was queried for MS FDA approved drugs and their pertaining drug targets. The searched drug targets were then compared to our resulting top 1 % modules and dual evaluation discovery modules. Overlapping drug targets and genes were then reported, along with corresponding p_g_ values.

## Results

### Multiple sclerosis modules identified by dmGWAS

**Fig. 2:**
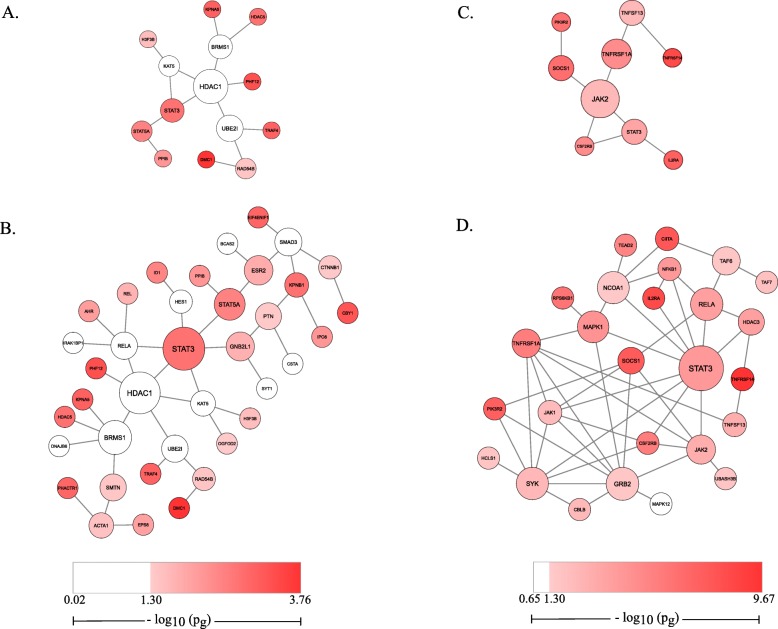
Top modules associated to GeneMSA GWAS and IMSGC GWAS. The top module of GeneMSA GWAS displays *HDAC1* as central (**a**). The top 10 modules of GeneMSA were merged (**b**). The top module of IMSGC GWAS shows *JAK2* as central (**c**). The top 10 modules of IMSGC were merged (**d**). Large nodes represent central genes and small nodes represent less central genes (based on measure of Betweenness). Colors of nodes represent Pascal reported gene-level *p*-values as specified by legend

We used dmGWAS tool to search the gene network modules with enriched signals in 2 MS GWAS datasets (Fig. 1). We identified 7458 network modules from GeneMSA GWAS data and 7566 modules from IMSGC GWAS data. We first examined those modules from GeneMSA data. The top module (the highest scored module) included 14 genes and had a normalized Z_m_ score 7.73 and module *p*-value (p_m_) of 1.04 × 10^− 14^. This top module had the central node *HDAC1* with a Betweenness measure of 0.76 and a gene-level *p*-value (p_g_) of 0.26 (Fig. 2a). The most significant genes within this top module included *PHF12* (p_g_ = 4.96 × 10^− 4^), which directly connected to central node *HDAC1*, and *DMC1* (p_g_ = 4.96 × 10^− 4^). Note that a network module with enriched association signals does not require each node to have significant p_g_ value; and this is one of the advantages to search a set of genes, each of which has weak or moderate signal, but their interaction contribute to a strong, combinatory signal. Among the 7458 modules identified from the GeneMSA GWAS dataset, the top 10 modules contained 37 genes. All top 10 modules had a normalized Z_m_ greater than 7.66 and a p_m_ less than 2.04 × 10^− 14^ (values pertaining to 10th top module). Cytoscape visualization of the merged GeneMSA top 10 modules (Fig. 2b) contained the central genes *STAT3* (p_g_ = 1.81 × 10^− 3^, Betweenness = 0.574) and *HDAC1* (p_g_ = 0.26, Betweenness = 0.54). Other central genes in GeneMSA top 10 modules included *STAT5A* (p_g_ = 3.25 × 10^− 3^, Betweenness = 0.28), *ESR2* (p_g_ = 0.02, Betweenness = 0.22) and *RELA* (p_g_ = 0.08, Betweenness = 0.16). Next, we merged the top 1 % modules (75 modules) to form a network. It contained 144 genes and presented *GNB2L1* (p_g_ = 0.02, Betweenness = 0.22), *SRC* (p_g_ = 0.03, Betweenness = 0.22) and *STAT3* (p_g_ = 1.81 × 10^− 3^, Betweenness = 0.20) as most central (Additional file 3: Figure S2).

The top module from the IMSGC GWAS dataset had a normalized Z_m_ value 11.13 (p_m_ < 2.20 × 10^− 16^) and contained 9 genes. *JAK2* was highly central (Betweenness = 0.75) within this top network with a p_g_ of 1.25 × 10^− 3^ (Fig. 2c). Highly significant genes within this top module were *TNFRSF14* (p_g_ = 2.16 × 10^− 10^) and *IL2RA* (p_g_ = 4.37 × 10^− 9^). Among the 7566 total modules identified from the IMSGC GWAS data, the top 10 modules contained 37 non-redundant genes. All these top 10 modules had Z_m_ values greater than 10.89 (p_m_ < 2.20 × 10^− 16^). When these top 10 modules were merged and evaluated by Cytoscape, the gene *STAT3* (p_g_ = 8.36 × 10^− 5^, Betweenness = 0.348) appeared central in this network (Fig. 2d). Other central genes in these top 10 modules included *GRB2* (p_g_ = 0.02, Betweenness = 0.16), *RELA* (p_g_ = 1.84 × 10^− 4^, Betweenness = 0.16), *SYK* (p_g_ = 3.41 × 10^− 3^, Betweenness = 0.16) and *NCOA1* (p_g_ = 0.03, Betweenness = 0.15). Next, we merged top 1 % modules (76 modules). The merged network included 123 genes and showed two highly central genes: *GRB2* (p_g_ = 0.02, Betweenness = 0.31) and *EGFR* (P_g_ = 0.07, Betweenness = 0.30) (Additional file 4: Figure S3).

**Fig. 3:**
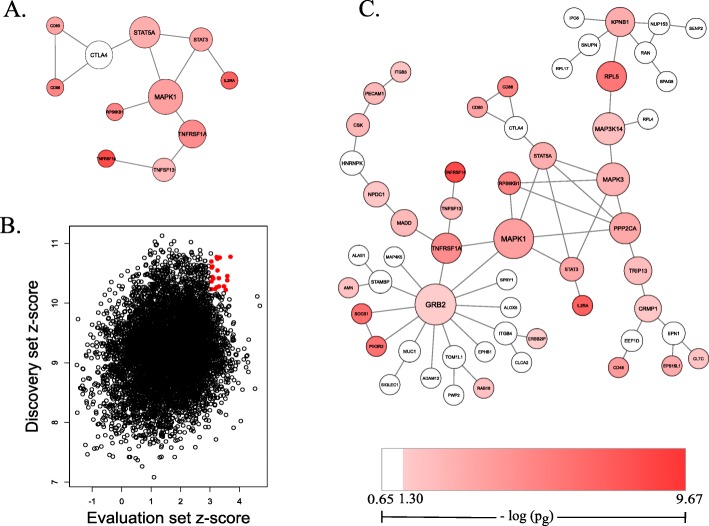
Significant modules of dual evaluation regarding IMSGC GWAS as discovery set. The top module of dual evaluation shows *MAPK1* as central (**a**). Modules are regarded as significant based on normalized evaluation and discovery z-scores. Significant modules are represented in red (**b**). The 20 significant modules were merged to show overall gene network (*n* = 56) (**c**). Large nodes represent central genes and small nodes represent less central genes (based on measure of Betweenness). Colors of nodes represent Pascal reported gene-level *p*-values as specified by legend

We next reported the dual evaluation results by using IMSGC GWAS as the discovery dataset and GeneMSA GWAS as the evaluation dataset. The top module (Fig. 3a) contained 11 genes and exhibited a normalized Z_m_ value 10.78 (p_m_ < 2.20 × 10^− 16^). This top module contained *MAPK1* (p_g_ = 3.40 × 10^− 5^) as a central node (Betweenness = 0.60). Two genes, *TNFRSF14* (p_g_ = 2.16 × 10^− 10^) and *IL2RA* (p_g_ = 4.37 × 10^− 9^), were highly significant and observed in the dual evaluation top module. In addition, dual evaluation of GeneMSA (evaluation) and IMSGC (discovery) datasets identified 20 significant modules based on discovery and evaluation Z_m_ values (Fig. 3b). All the 20 significant modules from the dual evaluation discovery set contained 56 non-redundant genes (Fig. 3c). The genes *GRB2* (p_g_ = 0.02, Betweenness = 0.54) and *MAPK1* (p_g_ = 3.40 × 10^− 5^, Betweenness = 0.51) were the most central genes in the merged network. Other central genes in this dual evaluation gene set included *MAPK3* (p_g_ = 5.07 × 10^− 4^, Betweenness = 0.33), *MAP3K14* (p_g_ = 3.78 × 10^− 3^, Betweenness = 0.31) and *PPP2CA* (p_g_ = 1.18 × 10^− 4^, Betweenness = 0.29).

### Gene set enrichment analysis

We first reported the functional enrichment analysis of gene list (*n* = 37 genes) from the top 10 modules of GeneMSA GWAS dataset (Additional file 5: Table S2). Interestingly, the most significantly enriched GO Molecular Function terms are all involved in the process of transcription regulation. These transcription regulation functions included “repressing transcription factor binding” (adjusted *p*-value = 2.79 × 10^− 7^) as the most enriched GO Molecular Function term, with six contributing genes: *CTNNB1, HDAC1, HDAC5, KAT5, RELA,* and *STAT3*. Among the top five enriched GO Biological Process terms, “regulation of glial cell differentiation” was enriched with an adjusted *p*-value of 2.58 × 10^− 3^. Contributing genes within the glial cell differentiation process included *CTNNB1, HDAC1, HES1* and *RELA*. Next, we reported the top five GO Cellular Component enriched terms. The second most significant one is “histone deacetylase complex” (adjusted *p*-value = 3.43 × 10^− 4^) with four contributing genes: *BRMS1, HDAC1, HDAC5* and *PHF12.* Lastly, the GO Cellular Component “I-kappaB/NF-kappaB complex” (adjusted *p*-value = 3.53 × 10^− 3^) was also among the top five enriched for GeneMSA, which included the input genes *REL* and *RELA*.

We next performed gene set enrichment analysis of the genes (*n* = 26 genes) from the top 10 modules from the IMSGC GWAS dataset (Additional file 6: Table S3). Among the enriched GO Molecular Function terms, the top term was “phosphotyrosine residue binding” with an adjusted *p*-value of 2.74 × 10^− 4^. This term was identified by the input genes *CBLB, GRB2* and *MAPK1*. Other top enriched GO Molecular Function terms of IMSGC included “protein phosphorylated amino acid binding” (adjusted *p*-value = 5.46 × 10^− 4^), “non-membrane spanning protein kinase activity” (adjusted *p*-value = 1.11 × 10^− 3^), “growth hormone receptor binding” (adjusted *p*-value = 1.11 × 10^− 3^) and “CCR5 chemokine receptor binding” (adjusted *p*-value = 1.11 × 10^− 3^). Among the enriched GO Biological Process terms of IMSGC, we found the “regulation of tyrosine phosphorylation of STAT protein” was the most significant (adjusted *p*-value = 1.58 × 10^− 5^). Five genes: *HCLS1, JAK2, SOCS1, STAT3*, and *TNFRSF1A* contributed to this GO term. Other STAT related processes within the top five IMSGC enriched GO Biological Process were “tyrosine phosphorylation of STAT protein” (adjusted *p*-value = 1.58 × 10^− 5^) and “positive regulation of STAT cascade” (adjusted *p*-value = 1.58 × 10^− 5^). The top GO Cellular Component term for IMSGC gene list was “I-kappaB/NF-kappaB complex” (adjusted *p*-value of 1.94 × 10^− 3^) with the contributing genes *NFKB1* and *RELA* (also observed as significant for the GeneMSA gene set).

Here, we reported the gene set enrichment analysis of the dual evaluation module genes (20 significant modules, 56 non-redundant genes, Table 1). Among the GO Molecular Function terms, the third most significant term is “virus receptor activity” (adjusted *p*-value of 1.67 × 10^− 3^), which was supported by four input genes: *CD80, CD86, ITGB3* and *TNFRSF14*. Phosphorylation and kinase related activity was once again observed within top five GO Molecular Function terms enriched for the dual evaluation gene set. These functions included “phosphotyrosine residue binding” (adjusted *p*-value = 1.67 × 10^− 3^), “phosphatidylinositol-4,5-bisphosphate 3-kinase activity” (adjusted *p*-value = 1.67 × 10^− 3^), “phosphatidylinositol bisphosphate kinase activity” (adjusted *p*-value = 1.67 × 10^− 3^), and “protein phosphorylated amino acid binding” (adjusted *p*-value = 1.67 × 10^− 3^). The top two enriched GO Biological Process terms in dual evaluation gene set were “T cell costimulation” (adjusted *p*-value = 2.11 × 10^− 6^) and “lymphocyte costimulation” (adjusted *p*-value = 2.11 × 10^− 6^)*,* which are at times necessary processes in activation of immune response [30, 31]. These costimulation processes both included the same 7 contributing genes: *CD80, CD86, CSK, CTLA4, GRB2, MAP3K14* and *TNFRSF14*. The “JAK-STAT cascade involved in growth hormone signaling” (adjusted *p*-value = 2.94 × 10^− 5^) was also present within top five enriched GO Biological Process terms of our dual evaluation. The top GO Cellular Component term enriched for this dual evaluation gene set was the “nuclear pore” with an adjusted *p*-value of 3.79 × 10^− 4^.

**Table 1:** Gene set enrichment analysis of the modules from dual evaluation^a^

GO term	# contributing genes/ term size^b^	Contributing genes^c^	*p*-value	adj. *p*-value^d^
Molecular Function				
Phosphotyrosine residue binding	3/18	*GRB2, MAPK1, MAPK3*	1.916 × 10^− 5^	1.674 × 10^− 3^
Phosphatidylinositol-4,5-bisphosphate 3-kinase activity	4/62	*CD80, CD86, GRB2, PIK3R2*	3.318 × 10^− 5^	1.674 × 10^− 3^
Virus receptor activity	4/71	*CD80, CD86, ITGB3, TNFRSF14*	5.667 × 10^−5^	1.674 × 10^−3^
Phosphatidylinositol bisphosphate kinase activity	4/72	*CD80, CD86, GRB2, PIK3R2*	5.987 × 10^−5^	1.674 × 10^−3^
Protein phosphorylated amino acid binding	3/28	*GRB2, MAPK1, MAPK2*	7.533 × 10^−5^	1.674 × 10^−3^
Biological Process				
T cell costimulation	7/82	*CD80, CD86, CSK, CTLA4, GRB2, MAP3K14, TNFRSF14*	3.704 × 10^−9^	2.113 × 10^−6^
Lymphocyte costimulation	7/83	*CD80, CD86, CSK, CTLA4, GRB2, MAP3K14, TNFRSF14*	4.037 × 10^−9^	2.113 × 10^−6^
JAK-STAT cascade involved in growth hormone signaling pathway	4/15	*MAPK1, MAPK3, STAT3, STAT5A*	8.415 × 10^−8^	2.937 × 10^−5^
Growth hormone receptor signaling pathway	4/24	*MAPK1, MAPK3, STAT3, STAT5A*	6.425 × 10^−7^	1.598 × 10^− 4^
Cellular response to growth hormone stimulus	4/25	*MAPK1, MAPK3, STAT3, STAT5A*	7.633 × 10^−7^	1.598 × 10^−4^
Cellular Component				
Nuclear pore	5/84	*KPNB1, NUP153, RAN, SENP2, SNUPN*	4.311 × 10^−6^	3.794 × 10^−4^
Clathrin-coated pit	4/69	*AMN, CLTC, EPN1, EPS15L1*	4.666 × 10^−5^	2.053 × 10^−3^
Endoplasmic reticulum tubular network	2/12	*KPNB1, RAB18*	5.296 × 10^−4^	0.0136
Platelet alpha granule membrane	2/13	*ITGB3, PECAM1*	6.248 × 10^−4^	0.0136
Cytosolic large ribosomal subunit	3/67	*RPL4, RPL5, RPL17*	9.556 × 10^−4^	0.0136

^a^In this dual evaluation, IMSGC GWAS was the discovery set and GeneMSA was the evaluation set

^b^Contributing genes: the number of genes in the input gene set. Term size: the total number of genes in the corresponding GO term

^c^Contributing genes: those in the input genes that contributed to the enrichment

^d^Adjusted *p*-value by the Benjamini-Hochberg method [28]

### Search for MS drug targets

MS FDA approved drugs and their drug targets were queried within DrugBank database for further assessment of the genes from our top networks. We found 32 unique MS FDA approved target genes. Among them, we found four drug targets overlapped with our top modules. These four genes are *HDAC1, IL2RA, KEAP1*, and *RELA*, which are the targets of 3 MS FDA approved drugs (Table 2). *HDAC1*, a gene in the histone deacetylase family and the central node in the top module of GeneMSA analysis (Fig. 2a), is a target gene of the MS drug fingolimod [29]. Note that *HDAC1* did not have a significant p_g_ value and would be missed in the typical GWAS analysis, but our network analysis could pinpoint this important druggable gene. Another MS drug target gene that overlapped within our modules was *IL2RA*, a gene which was present within the top module of IMSGC analysis, as well as in the dual evaluation top module. *IL2RA* is a target gene of the MS drug ocrelizumab. Lastly, the genes *RELA* and *KEAP1* are targets for the MS drug dimethyl fumarate [29]. *RELA* was present and relatively central within GeneMSA and IMSGC top 10 module gene lists (Fig. 2b, d). *KEAP1* was present within top 1 % modules of IMSGC (Additional file 4: Figure S3). Our network analysis indicated its potential to find potential druggable genes from GWAS datasets, which often reported significant variants in the non-coding regions.

**Table 2:** MS FDA drug target genes within top modules

Drug name	Commercial product name	Target gene	Occurrence in top modules	GeneMSA gene-level *p*-value	IMSGC gene-level *p*-value
*Fingolimod*	Gilenya	*HDAC1*	Central in top module of GeneMSA, present in 75/75 top 1 % GeneMSA modules	0.263	0.249
*Ocrelizumab*	Ocrevus	*IL2RA*	Present in top modules of IMSGC GWAS and top module of dual evaluation, present in 76/76 top 1 % IMSGC modules	0.212	4.37 × 10^−9^
*Dimethyl fumarate*^a^	Tecfidera	*KEAP1*	Present in 1/76 IMSGC top one present modules	0.172	2.22 × 10^−6^
		*RELA*	Present in 3/75 top 1 % GeneMSA modules and 7/76 IMSGC top 1 % modules	0.084	1.84 × 10^−4^

^a^*Dimethyl fumarate* has two target genes from module gene list (*KEAP1* and *RELA*)

## Discussion

Network approaches can assist the original GWAS analysis to find combinatory signals in a set of genes that are functionally related. This approach will help better interpret the genetic association results toward understanding the molecular mechanisms of the complex disease. In this study, the most significant modules identified by dmGWAS contained central genes that were not addressed in the original GWAS reports. This is mainly due to the genome-wide significance level set in the original GWA studies (e.g. *p* value < 10^− 8^ for each SNP). Central genes outside of significance levels may have been revealed as important interactors for their connections with highly associated genes. Four unique MS drug target genes were included in our top modules: *HDAC1, IL2RA, KEAP1* and *RELA*. The drug target genes *HDAC1* and *RELA* in particular were central in top modules and also contributing genes in several notable gene set enrichment results relevant to hypothesized MS mechanisms. Furthermore, *GRB2, JAK2, MAPK1* and *STAT3* are genes of interest reported based on centrality measures. Common themes relevant to MS observed within our gene set enrichment analysis include immune system pathways, transcription and histone modification pathways, as well as growth hormone signaling pathways. Hypothesis for transcription dysregulation and epigenetic factors in MS were supported. Apart from these common themes, other pertinent enriched functions include the I-kappaB/NF-kappa B complex, glial cell differentiation, virus receptor activity and T-cell and lymphocyte costimulation. These enriched GO terms supported the inflammation and viral infection onset hypotheses for MS, which have not been reported in original GWA studies.

### Several genes of interest are FDA-approved MS drug target genes

The genes *HDAC1, IL2RA, KEAP1* and *RELA* present within our top gene networks are of interest, as they are drug-target genes for FDA approved MS disease-modifying therapies. Gene *HDAC1,* which encodes histone deacetylase 1, was the most central gene in the top module of the GeneMSA GWAS dataset (Fig. 2a) and remained one of the most central genes in merged top 10 modules and merged top 1 % modules (Fig. 2b, Additional file 3: Figure S2). Although *HDAC1* is outside of the accepted significance level, it may have been presented as central for being an important interactor with the relatively significant gene *PHF12* and other relevant genes. It has been suggested that *HDAC1* plays a role in MS pathogenesis. *HDAC1* modulates histone acetylation, which plays a role in the epigenetic control of T-cell mediated immunity. Mice with a conditional deletion of *HDAC1* have shown to be resistant to experimental autoimmune encephalomyelitis (EAE), a common animal model of MS [32]. Moreover, nuclear export of *HDAC1* is associated with impaired mitochondrial function and axonal damage in MS [33]. *HDAC1* is a drug-target gene for fingolimod [29, 34] a sphingosine 1-phosphate (S1P) receptor modulator which is thought to mediate its effect in MS by sequestering lymphocytes within secondary lymphoid tissues [35]. The structure of the active metabolite of fingolimod mimics that of S1P, a signaling lipid involved in many cellular functions. It has been shown that fingolimod-P, produced within the nucleus from the phosphorylation of fingolimod, binds and inhibits class I HDAC, including *HDAC1*, consequently enhancing specific histone acetylation [36]. This effect is carried out independently of S1P receptors.

The gene *RELA* is also a gene of interest, as it is relatively central in top modules of GeneMSA and IMSGC (Fig. 2b, d), though it was not reported by either GWAS study examined. *RELA* is a proto-oncogene that encodes for a subunit of NF-kappaB. NF-kappaB is thought to play a central role in MS as it regulates innate and adaptive immunity, and is involved in the activation of astrocytes [37, 38]. Activation of NF-kappaB has been described in MS brain tissue [39]. Transgenic inhibition of astroglial NF-kappaB was shown to improve functional outcome in EAE [40]. *RELA* has been identified as a drug-target gene of the MS medication dimethyl fumarate. Indeed, monomethyl fumarate, the active metabolite of dimethyl fumarate, was shown to decrease *RELA* expression in myeloid dendritic cells derived from MS patients [41].

*IL2RA* and *KEAP1* are another two genes of interest within our top gene networks because they are drug target genes in MS. *IL2RA* is a drug-target gene of ocrelizumad, a recently FDA approved drug for treatment of both relapsing-remitting and primary progressive MS, and was present within our top gene module of IMSGC (Fig. 2c) and the top gene module of our dual evaluation (Fig. 3a). Ocrelizumab is an anti-CD20 monoclonal antibody. Its efficacy in MS is attributed to B-cell depletion. *IL2RA* encodes for a protein that is a constituent of the IL2 receptor. It was reported by the IMSGC GWAS to be a genetic risk factor of MS. Lastly, although *KEAP1* was not reported by either GWAS study, *KEAP1* is a drug target gene of the MS drug dimethyl fumarate and was present within one of our top 1 % IMSGC modules. The gene *KEAP1* encodes for an adapter protein of the E3 ubiquitin ligase, which are involved in DNA repair and cell cycle controls. Dimethyl fumarate, the drug which targets *RELA* and *KEAP1*, is an anti-inflammatory and central nervous system agent [29]. Although it is known that dimethyl fumarate upregulates nuclear-derived 2 (Nrf2) pathway, its relevance to MS is only hypothesized [29].

### Additional genes of interest may be promising drug targets

Additional genes of interest were highlighted by their centrality within top gene modules: *GRB2, JAK2, MAPK1* and *STAT3*. Given that the central genes *HDAC1* and *RELA* genes are currently drug targets of FDA approved MS treatment, this suggests other central genes identified may be promising drug targets for future investigation. The protein encoding gene *JAK2*, which produces a protein that promotes cell proliferation through the JAK/STAT pathway, was central in our top gene module of IMSGC GWAS dataset (Fig. 2c). This gene was not previously reported by either MS GWA study. The gene *STAT3,* which was central in merged top 10 modules of GeneMSA and IMSGC and encodes for a protein that is a member of the STAT protein family, also plays a key role in the JAK-STAT signaling pathway. *STAT3* was previously reported by the IMSGC GWAS and polymorphisms of *STAT3* have been considered risk factors of MS, although the mechanism by which this association occurs is not fully understood [42, 43]. Previous work has highlighted the role of the JAK/STAT signaling pathway in the regulation of innate immunity and promotion of oligodendrocyte apoptosis and demyelination [44]. Cytokines and oxidative stress promote inflammation-related gene expression via *JAK2* phosphorylation and *STAT3* transcription factor activation [45]. Liu et al. demonstrated that *JAK2* inhibitors have clinical efficacy in multiple preclinical models of MS by suppressing downstream activation of STAT, particularly *STAT3* [46]. Modulation of the JAK/STAT pathway is thought to mediate the beneficial effect of neuroprotective compound linagliptin in cuprizone-induced demyelination [47]. Studies have suggested that JAK/STAT signaling occurs upstream of NF-kappaB activation [48]. Further evidence for the involvement of the JAK/STAT and NF-kappaB pathways in MS pathogenesis was obtained from our gene set enrichment analyses. Regulation of tyrosine phosphorylation of STAT protein, which included *JAK2* and *STAT3* was also observed within top gene enrichment of IMSGC, along with more STAT regulation pathways. Within the dual evaluation enrichment results, the JAK-STAT cascade involved in growth hormone signaling pathway was observed with the contributing genes of interest *MAPK1* and *STAT3*. The cellular component I-kappaB/NF-kappaB complex was enriched for both the GeneMSA and IMSGC enrichment results and included the contributing drug target gene *RELA.*

*MAPK1,* a gene part of the MAP kinase family and an essential part of the growth hormone signaling cascade, was the central gene in the top module of our dual evaluation (Fig. 3a), regarding IMSGC as the discovery set. *MAPK1* was previously reported by the IMSGC GWAS, and has been identified as a risk locus for MS [49]. The gene *GRB2*, a gene that encodes for the growth factor receptor bound protein, was seen as central in the merged significant dual evaluation gene modules (Fig. 3c) and in the top 1 % modules of IMSGC (Additional file 4: Figure S3). This gene was not reported by either GWA study examined. However, *GRB2* has also been considered of a biological relevance by another MS network-based report and has been shown to be upregulated in EAE [50, 51]. The genes *GRB2, JAK2, MAPK1* and *STAT3* may be of consideration for future MS drug target experimentation because of their central presence in our top modules.

### Gene set enrichment analysis supports epigenetic hypothesis in MS

Transcription factors have been attributed to subtypes of MS and hypothesized to dysregulate certain genes, thereby contributing to MS onset [52, 53]. The involvement of transcription factor dysregulation is supported by our study, given that our enrichment results showed many transcription factor processes within top five significantly enriched categories. The top enriched GO Molecular Function term in GeneMSA was “repressing transcription factor binding”, which included the genes of interest *HDAC1, RELA* and *STAT3*. Other enriched transcription regulation functions reported for GeneMSA include “activating transcription factor binding” and “RNA polymerase II distal enhancer sequence-specific DNA binding”, which both included *HDAC1* and *RELA*. Transcription regulation GO Cellular Component terms enriched for IMSGC were “transcription factor TFTC complex” and “transcription factor TFIID”. Additionally, epigenetic factors are thought to mitigate the environmental factors of MS [34]. This epigenetic factor hypothesis of MS was also supported by our results, as “histone deacetylase complex” was a top enriched GO Molecular Function term and a member of the histone deacetylase family, *HDAC1*, was highly central in top GeneMSA modules [34]. Another epigenetic enriched function within GeneMSA was the GO Molecular Function term “chromatin DNA binding”, which included the genes of interest *HDAC1, RELA* and *STAT3*.

### Genes identified for enriched regulation of glial cell differentiation and growth factor signaling

Regulation of glial cell differentiation was observed for the GeneMSA enrichment results. Glial cells include oligodendrocytes, astrocytes, and microglia, which are cells of interest in processes of MS pathogenesis. Oligodendrocytes are cells responsible for myelin sheath synthesis, and microglia and astrocytes are involved in innate immune responses within the central nervous system [54–56]. Myelin restoration is seen as a therapeutic strategy that could potentially aid in reversing neurological disabilities exhibited by people living with MS [57, 58]. As MS remyelinating therapies become more sought after, this suggests that genes involved in the enriched function for regulation of glial cells may be of interest: *CTNNB1, HDAC1, HES1* and *RELA.* Interestingly, epidermal growth factor receptor (*EGFR*) was shown as central within our top 1 % IMSGC gene modules (Additional file 4: Figure S3), and involvement of growth factors signaling pathways, phosphotyrosine residue binding and protein phosphorylated amino acid binding are supported by our gene set enrichment analysis. *EGFR* overexpression was found to enhance oligodendrocyte differentiation and axonal myelination, suggesting that *EGFR* targeting may represent a viable strategy for myelin repair [57].

### Viral insult hypothesis for MS etiology supported by virus receptor activity and T cell costimulation

Although the cause of MS is unknown, this complex disease has been attributed to the occurrence of a viral insult at a young age, later causing onset of neurological deficits in genetically predisposed individuals. This hypothesis is regarded as the most probable by some investigators [31]. Functional enrichment analysis of significant dual evaluation gene modules supports this hypothesis as T cell costimulation, lymphocyte costimulation and virus receptor activity were significantly enriched. Costimulation pathways of T cell and B cell activation have been exhibited in pathogen infection, and costimulation pathways of immune response activation have been linked to a viral context [30]. These lymphocyte costimulation pathways, coupled with enrichment of virus receptor activity suggest that the viral hypothesis for MS onset is relevant, and may occur through T cell costimulation. The genes found to be involved in MS virus receptor activity may be of interest for experimental validation of this viral onset hypothesis: *CD80, CD86, ITGB3* and *TNFRSF14.* Finally, the genes involved in T-cell costimulation may also be further investigated to discover if the autoimmune response in MS truly occurs through this immune response activation pathway: *CD80, CD86, CSK, CTLA4, GRB2, MAP3K14* and *TNFRSF14.*

### Limitations and future work

There are several limitations in this study. One is data heterogeneity between IMSGC and GeneMSA GWAS datasets. Another limitation of this study is that some genes within our networks may be highly connected with the most significant genes (at individual gene level), and this makes them more likely to be detected due to lack of PPI reference network completeness. On the other hand, the PPI network is still incomplete, and does not include all the protein-coding genes. Thirdly, the highly associated HLA region within MS GWAS datasets is also a limiting factor, as these highly associated regions may mask the association signals of other regions. We will expand our work in future. First, we may include more genetic association data, including both common and rare variants from genome sequencing data, once they are available. Second, we may integrate gene expression and GWAS signal from the same disease (MS) to detect more reliable and causal signals. Tools such as EW-dmGWAS [59] can be applied. Thirdly, functional annotations are available from various resources. We will evaluate these association signals using additional annotations such as epigenetic marks, expression quantitative trait loci (eQTL), tissue-specificity and cell types, among others. Tissue specificity enrichment analysis of genetic variations in association studies has shown interesting results, as demonstrated in our multi-trait GWAS analysis [60]. Finally, further experimental validation of supported and proposed MS mechanisms is needed using cell lines and animal models.

## Conclusions

The dmGWAS (version 2.4) tool, previously developed by members of our team, was used to integrate the GeneMSA and IMSGC GWAS with the human interactome to interpret genetic associations. Through a dual evaluation approach, this network-based analysis could effectively yield top modules evaluated by the presence of MS FDA-approved drug target genes: *HDAC1, IL2RA, KEAP1* and *RELA*. Other central genes present within top modules are suggested as potential drug targets for MS: *GRB2, JAK2, MAPK1* and *STAT3*. The independent gene set enrichment results of both GeneMSA and IMSGC top modules supported the hypothesis of epigenetic factors involved in dysregulation of genes in MS. GeneMSA enrichment of top 10 modules presented a pathway involved in regulation of glial cell differentiation, thereby identifying genes that are promising for remyelination therapy investigations. Furthermore, a dual evaluation of both MS GWAS identified genes enriched in virus receptor activity, supporting the viral onset hypothesis for the etiology of MS.

## Supplementary information

**Additional file 1: Table S1.** Publicly available MS GWAS summary statistics data.

**Additional file 2: Figure S1.** SNP-level and gene-level Manhattan plots comparing individual MS GWAS data.

**Additional file 3: Figure S2.** Top 1% modules of GeneMSA.

**Additional file 4: Figure S3.** Top 1% modules of IMSGC.

**Additional file 5: Table S2.** Gene set enrichment analysis of modules from dual evaluation of GeneMSA.

**Additional file 6: Table S3.** Gene set enrichment analysis of modules from dual evaluation of IMSGC.

## Data Availability

All the data used in this study are from public sources cited in our reference list. Also, additional files, which may be needed to reproduce the results presented in the manuscript, are made available as additional files.
