# Supplementary material for: Dense module searching for gene networks associated with multiple sclerosis
Source: BMC Med Genomics. 2020 Apr 3;13(Suppl 5):48. doi: 10.1186/s12920-020-0674-5 (PMC7118851; doi:10.1186/s12920-020-0674-5)
Supplement: Supplementary file 1 — Additional file 1: Table S1. Publicly available MS GWAS summary statistics data. [file 12920_2020_674_MOESM1_ESM.docx]

**Table S1: Publicly available MS GWAS summary statistics data**

| GWAS dataset | Data accession | Study type | Population | Genotyping platform | Sample sizes | SNP count |
| --- | --- | --- | --- | --- | --- | --- |
| GeneMSA | dbGaP | Case Control | Northern European | Sentrix HumanHap550- BeadChip | 978 cases,  883 controls | 514599 |
| IMSGC | GWAS Catalog | Case Control | European | Human660-Quad chip | 9,772 cases,  17,376 controls | 472087 |
