# Supplementary material for: Dense module searching for gene networks associated with multiple sclerosis
Source: BMC Med Genomics. 2020 Apr 3;13(Suppl 5):48. doi: 10.1186/s12920-020-0674-5 (PMC7118851; doi:10.1186/s12920-020-0674-5)
Supplement: Supplementary file 2 — Additional file 2: Figure S1. SNP-level and gene-level Manhattan plots comparing individual MS GWAS data. [file 12920_2020_674_MOESM2_ESM.pdf]

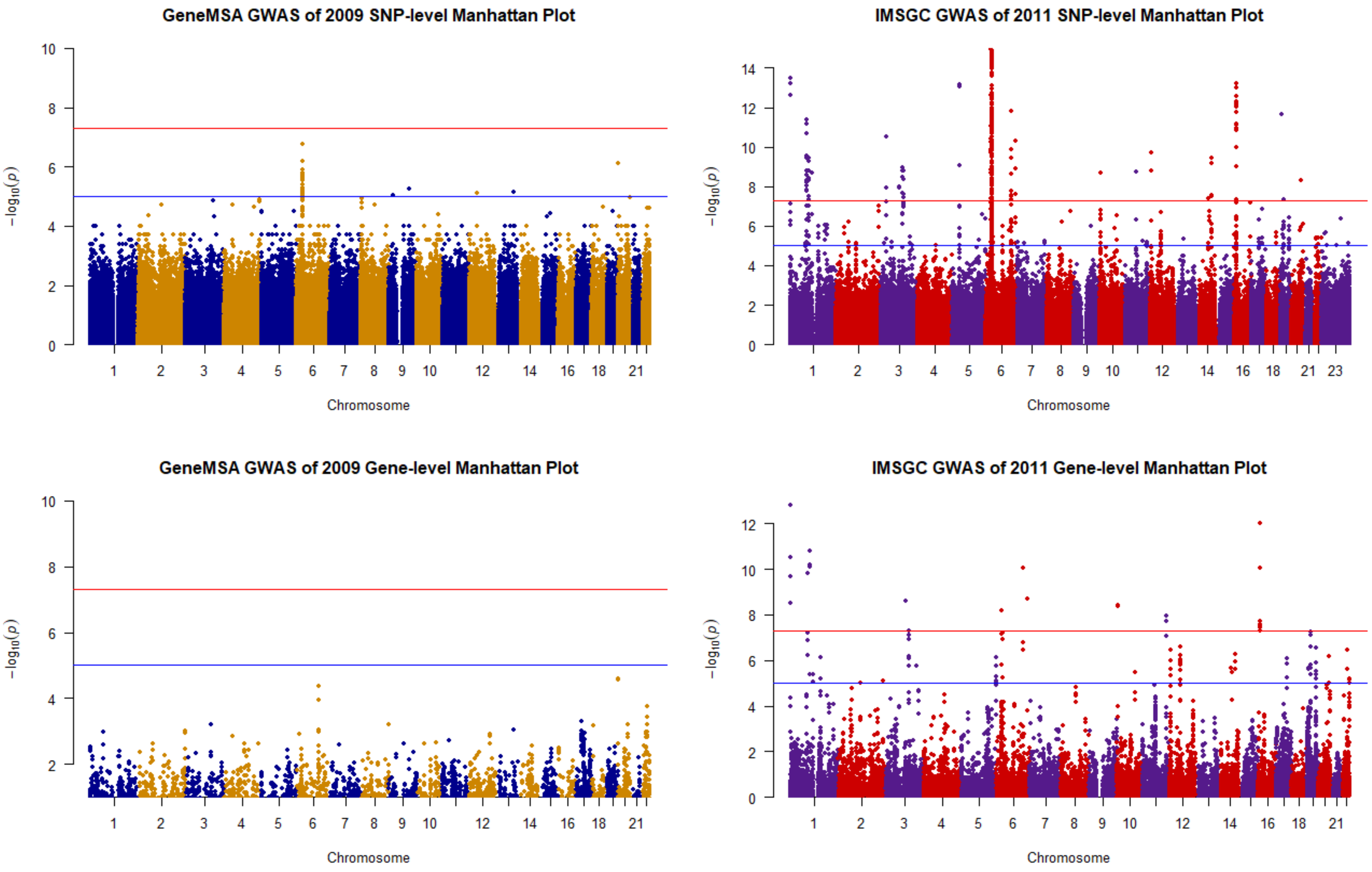
GeneMSA GWAS of 2009 SNP-level Manhattan Plot
IMSGC GWAS of 2011 SNP-level Manhattan Plot
GeneMSA GWAS of 2009 Gene-level Manhattan Plot
IMSGC GWAS of 2011 Gene-level Manhattan Plot
$-\log_{10}(p)$
Chromosome
0
2
4
6
8
10
12
14
1
2
3
4
5
6
7
8
9
10
12
14
16
18
21
23
