# Supplementary figures and images for: Dense module searching for gene networks associated with multiple sclerosis
Source: BMC Med Genomics. 2020 Apr 3;13(Suppl 5):48. doi: 10.1186/s12920-020-0674-5 (PMC7118851; doi:10.1186/s12920-020-0674-5)

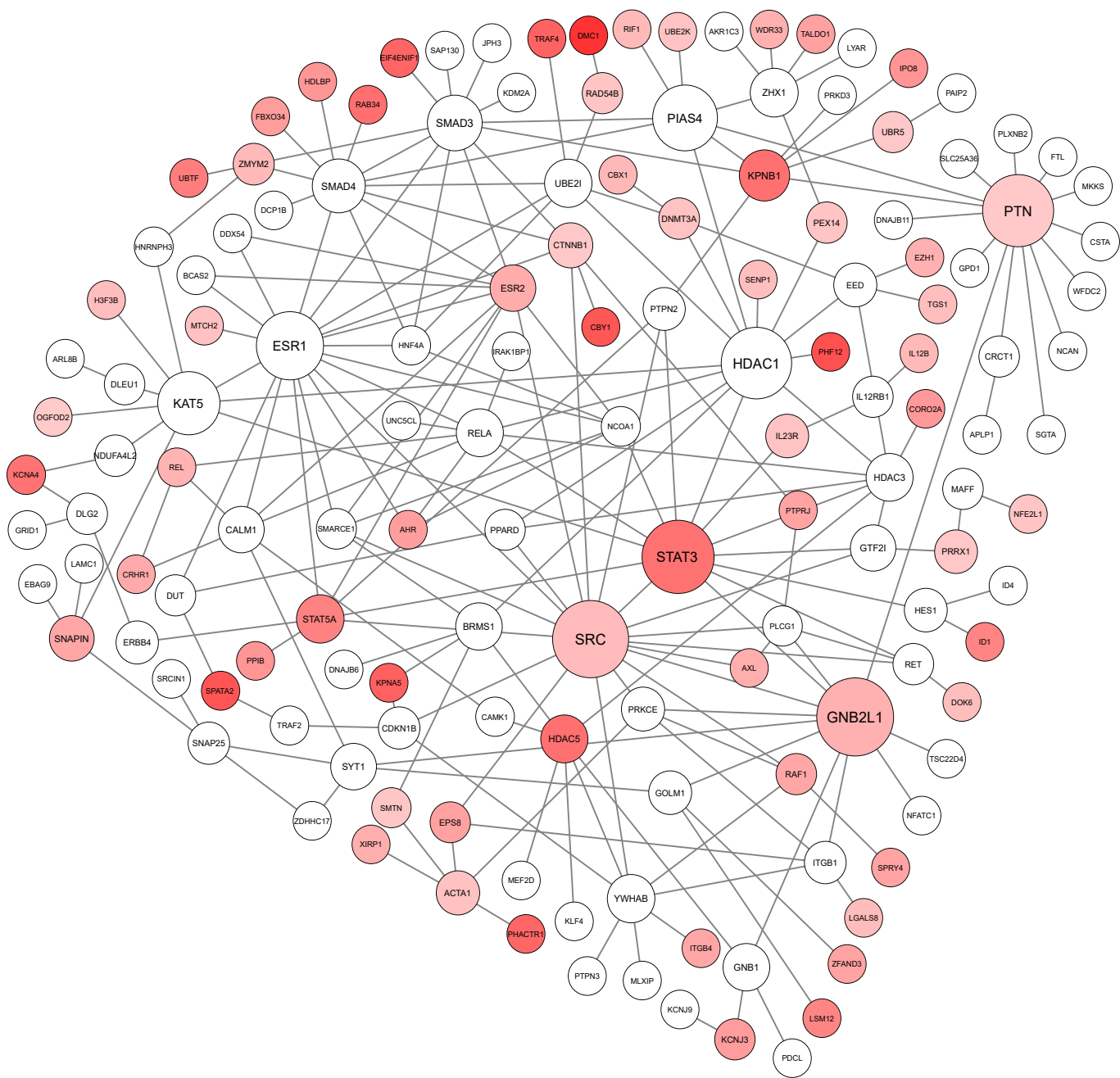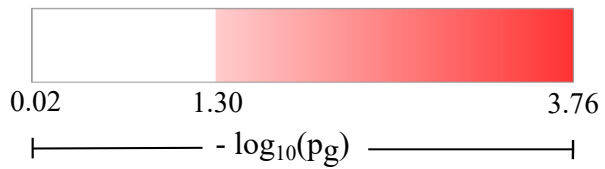

Supplement: Supplementary file 3 — Additional file 3: Figure S2. Top 1% modules of GeneMSA. [file 12920_2020_674_MOESM3_ESM.pdf]

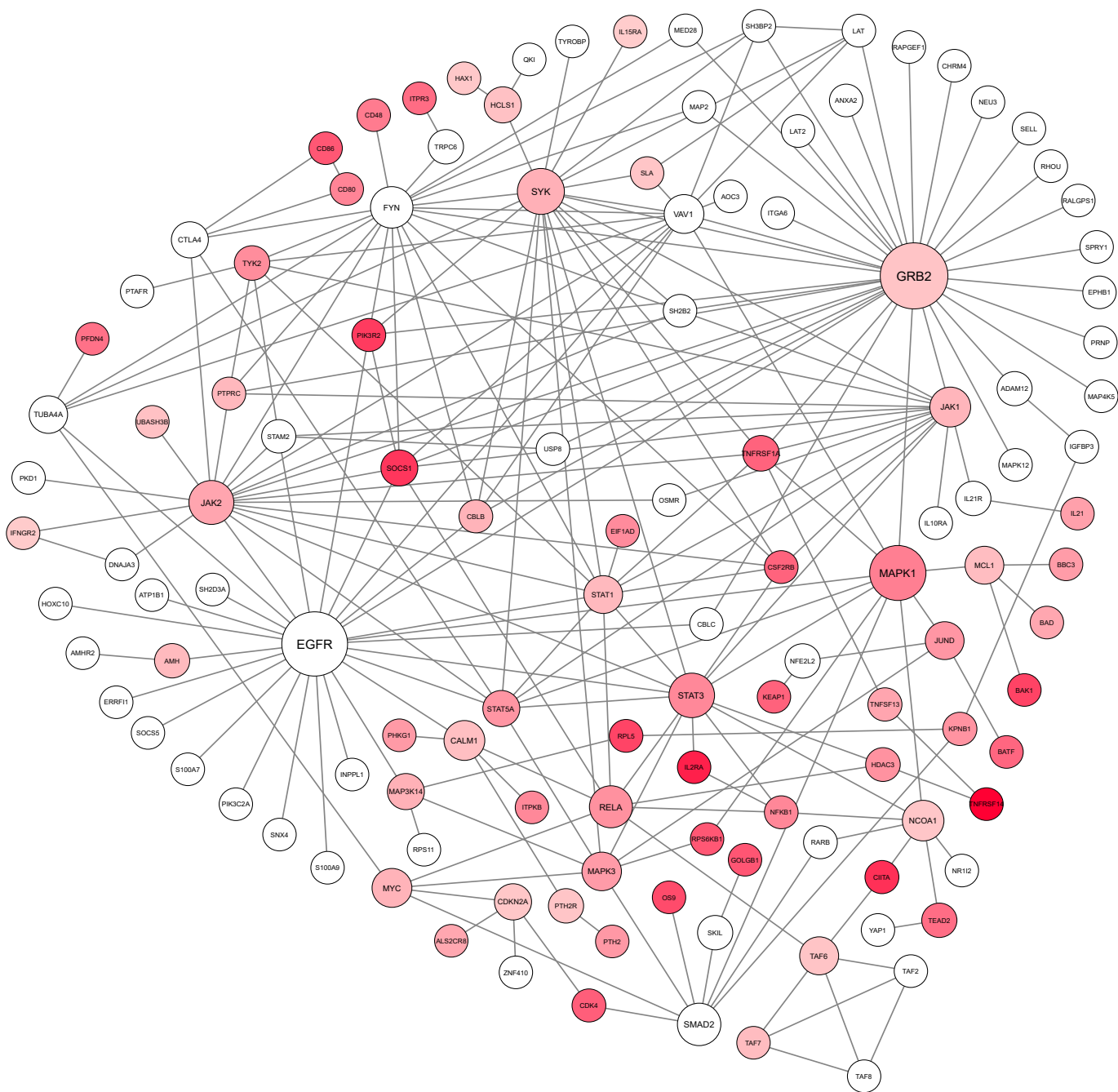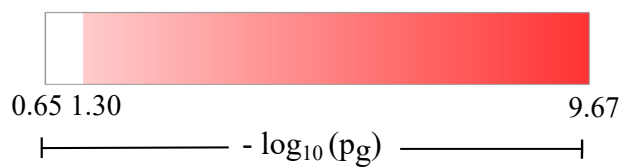

Supplement: Supplementary file 4 — Additional file 4: Figure S3. Top 1% modules of IMSGC. [file 12920_2020_674_MOESM4_ESM.pdf]
