# Supplementary material for: Dense module searching for gene networks associated with multiple sclerosis
Source: BMC Med Genomics. 2020 Apr 3;13(Suppl 5):48. doi: 10.1186/s12920-020-0674-5 (PMC7118851; doi:10.1186/s12920-020-0674-5)
Supplement: Supplementary file 5 — Additional file 5: Table S2. Gene set enrichment analysis of modules from dual evaluation of GeneMSA. [file 12920_2020_674_MOESM5_ESM.docx]

**Table S2: Gene set enrichment analysis of modules from dual evaluation of GeneMSA**

| GO term | # contributing genes/ term size^a^ | Contributing genes^b^ | p-value | adj. p-value^c^ |
| --- | --- | --- | --- | --- |
| Molecular Function | | | | |
| Repressing transcription factor binding | 6/60 | CTNNB1, HDAC1, HDAC5, KAT5, RELA, STAT3 | 1.839×10^-9^ | 2.758×10^-7^ |
| Chromatin DNA binding | 5/85 | H3F3B, HDAC1, RELA, SMAD3, STAT3 | 6.766×10^-7^ | 5.074×10^-5^ |
| Activating transcription factor binding | 4/61 | CTNNB1, HDAC1, RELA, SMAD3 | 6.295×10^-6^ | 3.147×10^-4^ |
| RNA polymerase II distal enhancer sequence-specific DNA binding | 4/71 | H3F3B, HDAC1, REL, RELA | 1.156×10^-5^ | 4.333×10^-4^ |
| Enhancer sequence-specific DNA binding | 9/93 | H3F3B, HDAC1, REL, RELA | 3.366×10^-5^ | 1.010×10^-3^ |
| Biological Process |  |  |  |  |
| Modification of morphology or physiology of other organism involved in symbiotic interaction | 5/96 | HDAC1, KPNA5, KPNB1, PPIB, SMAD3 | 1.253*×*10^-6^ | 1.492*×*10^-3^ |
| Epithelial cell differentiation involved in prostate gland development | 3/15 | CTNNB1, ESR2, STAT5A | 3.231*×*10^-6^ | 1.916*×*10^-3^ |
| Prostate gland development | 4/57 | AHR, CTNNB1, ESR2, STAT5A | 4.830*×*10^-6^ | 1.916*×*10^-3^ |
| Androgen receptor signaling pathway | 4/65 | CTNNB1, ESR2, HDAC1, KAT5 | 8.185*×*10^-6^ | 2.435*×*10^-3^ |
| Regulation of glial cell differentiation | 4/71 | CTNNB1, HES1, HDAC1, RELA | 1.165*×*10^-5^ | 2.580*×*10^-3^ |
| Cellular Component | | | | |
| Sin3-type complex | 3/17 | BRMS1, HDAC1, PHF12 | 4.493*×*10^-6^ | 3.429*×*10^-4^ |
| Histone deacetylase complex | 4/63 | BRMS1, HDAC1, HDAC5, PHF12 | 6.593*×*10^-6^ | 3.429*×*10^-4^ |
| I-kappaB/NF-kappaB complex | 2/8 | REL, RELA | 1.019*×*10^-4^ | 3.533*×*10^-3^ |
| Sin3 complex | 2/14 | HDAC1, PHF12 | 3.288*×*10^-4^ | 8.548*×*10^-3^ |
| Transcriptional repressor complex | 3/79 | HDAC1, HDAC5, PHF12 | 4.810*×*10^-4^ | 0.01 |

^a^ Contributing genes: the number of genes in the input gene set. Term size: the total number of genes in the corresponding GO term.

^b^ Contributing genes: those in the input genes that contributed to the enrichment.

^c^ Adjusted p-value by the Benjamini-Hochberg method [27]
