# Supplementary material for: Dense module searching for gene networks associated with multiple sclerosis
Source: BMC Med Genomics. 2020 Apr 3;13(Suppl 5):48. doi: 10.1186/s12920-020-0674-5 (PMC7118851; doi:10.1186/s12920-020-0674-5)
Supplement: Supplementary file 6 — Additional file 6: Table S3. Gene set enrichment analysis of modules from dual evaluation of IMSGC. [file 12920_2020_674_MOESM6_ESM.docx]

**Table S3: Gene set enrichment analysis of modules from dual evaluation of IMSGC**

| GO term | # contributing genes/ term size^a^ | Contributing genes^b^ | p-value | adj. p-value^c^ |
| --- | --- | --- | --- | --- |
| Molecular Function | | | | |
| Phosphotyrosine residue binding | 3/18 | CBLB, GRB2, MAPK1 | 1.932×10^-6^ | 2.744×10^-4^ |
| Protein phosphorylated amino acid binding | 3/28 | CBLB, GRB2, MAPK1 | 7.686×10^-6^ | 5.457×10^-4^ |
| Growth hormone receptor binding | 2/6 | JAK1, JAK2 | 2.790×10^-5^ | 1.109×10^-3^ |
| Non-membrane spanning protein tyrosine kinase activity | 3/46 | JAK1, JAK2, SYK | 3.503×10^-5^ | 1.109×10^-3^ |
| CCR5 chemokine receptor binding | 2/7 | JAK1, STAT3 | 3.903×10^-5^ | 1.109×10^-3^ |
| Biological Process |  |  |  |  |
| Regulation of tyrosine phosphorylation of STAT protein | 5/77 | JAK2, HCLS1, SOCS1, STAT3, TNFRSF1A | 6.510×10^-8^ | 1.582×10^-5^ |
| Tyrosine phosphorylation of STAT protein | 5/81 | JAK2, HCLS1, SOCS1, STAT3, TNFRSF1A | 8.410×10^-8^ | 1.582×10^-5^ |
| Cellular response to interleukin-3 | 3/7 | CSF2RB, JAK1, JAK2 | 8.424×10^-8^ | 1.582×10^-5^ |
| Response to interleukin-3 | 3/7 | CSF2RB, JAK1, JAK2 | 8.424×10^-8^ | 1.582×10^-5^ |
| Positive regulation of STAT cascade | 5/86 | JAK2, HCLS1, SOCS1, STAT3, TNFRSF1A | 1.138×10^-7^ | 1.582×10^-5^ |
| Cellular Component | | | | |
| I-kappaB/NF-kappaB complex | 2/8 | NFKB1, RELA | 4.984×10^-5^ | 1.944×10^-3^ |
| transcription factor TFTC complex | 2/14 | TAF6, TAF7 | 1.612×10^-4^ | 3.143×10^-3^ |
| transcription factor TFIID complex | 2/23 | TAF6, TAF7 | 4.447×10^-4^ | 5.161×10^-3^ |
| MLL1 complex | 2/28 | TAF6, TAF7 | 6.617×10^-4^ | 5.161×10^-3^ |
| MLL1/2 complex | 2/28 | TAF6, TAF7 | 6.617×10^-4^ | 5.161×10^-3^ |

^a^ Contributing genes: the number of genes in the input gene set. Term size: the total number of genes in the corresponding GO term.

^b^ Contributing genes: those in the input genes that contributed to the enrichment.

^c^ Adjusted p-value by the Benjamini-Hochberg method [27]
